# Total venous coronary artery bypass grafting for acute myocardial infarction with Leriche syndrome and porcelain aorta: a case report

**DOI:** 10.1093/jscr/rjaf1102

**Published:** 2026-01-20

**Authors:** Hideki Isa, Kentaro Shirakura, Tasuku Kawarabayashi, Hidenobu Akamatsu, Koji Kagawa, Kazuki Miyatani, Nobuhiro Mochizuki, Fumitaka Suzuki, Ryohei Ushioda, Ryo Okubo, Aina Hirofuji, Shingo Kunioka, Masahiro Tsutsui, Hiroyuki Kamiya

**Affiliations:** Department of Cardiac Surgery, Asahikawa Medical University, Midorigaoka Higashi 2-1-1-1, Asahikawa 078-8510, Japan; Department of Cardiovascular Surgery, Nayoro City General Hospital 1 Nishinanajo Minami 8-chome, Nayoro, Hokkaido 096-8511, Japan; Department of Cardiac Surgery, Asahikawa Medical University, Midorigaoka Higashi 2-1-1-1, Asahikawa 078-8510, Japan; Department of Cardiovascular Surgery, Nayoro City General Hospital 1 Nishinanajo Minami 8-chome, Nayoro, Hokkaido 096-8511, Japan; Department of Cardiac Surgery, Asahikawa Medical University, Midorigaoka Higashi 2-1-1-1, Asahikawa 078-8510, Japan; Department of Cardiovascular Surgery, Nayoro City General Hospital 1 Nishinanajo Minami 8-chome, Nayoro, Hokkaido 096-8511, Japan; Department of Cardiac Surgery, Asahikawa Medical University, Midorigaoka Higashi 2-1-1-1, Asahikawa 078-8510, Japan; Department of Cardiovascular Surgery, Nayoro City General Hospital 1 Nishinanajo Minami 8-chome, Nayoro, Hokkaido 096-8511, Japan; Department of Cardiac Surgery, Asahikawa Medical University, Midorigaoka Higashi 2-1-1-1, Asahikawa 078-8510, Japan; Department of Cardiac Surgery, Asahikawa Medical University, Midorigaoka Higashi 2-1-1-1, Asahikawa 078-8510, Japan; Department of Cardiac Surgery, Asahikawa Medical University, Midorigaoka Higashi 2-1-1-1, Asahikawa 078-8510, Japan; Department of Cardiac Surgery, Asahikawa Medical University, Midorigaoka Higashi 2-1-1-1, Asahikawa 078-8510, Japan; Department of Cardiac Surgery, Asahikawa Medical University, Midorigaoka Higashi 2-1-1-1, Asahikawa 078-8510, Japan; Department of Cardiac Surgery, Asahikawa Medical University, Midorigaoka Higashi 2-1-1-1, Asahikawa 078-8510, Japan; Department of Cardiac Surgery, Asahikawa Medical University, Midorigaoka Higashi 2-1-1-1, Asahikawa 078-8510, Japan; Department of Cardiac Surgery, Asahikawa Medical University, Midorigaoka Higashi 2-1-1-1, Asahikawa 078-8510, Japan; Department of Cardiac Surgery, Asahikawa Medical University, Midorigaoka Higashi 2-1-1-1, Asahikawa 078-8510, Japan; Department of Cardiac Surgery, Asahikawa Medical University, Midorigaoka Higashi 2-1-1-1, Asahikawa 078-8510, Japan

**Keywords:** Leriche syndrome, coronary artery bypass grafting, ischemic heart disease, coronary revascularization

## Abstract

Leriche syndrome is an occlusive disease involving the abdominal aorta and iliac arteries that is frequently associated with ischemic heart disease (IHD). The internal thoracic arteries in patients with Leriche syndrome often serve as important collateral pathways to the lower extremities. Therefore, graft selection for coronary artery bypass grafting (CABG) requires special consideration. We performed on-pump total venous CABG in a patient with acute heart failure secondary to IHD complicated by Leriche syndrome who required emergent coronary revascularization. This strategy was chosen to preserve lower limb perfusion. When coronary revascularization is a priority for patients with IHD complicated by Leriche syndrome, on-pump total venous CABG is a reasonable option because of the need to preserve lower limb perfusion and difficulty of using mechanical circulatory support devices for occlusive disease.

## Introduction

Leriche syndrome is an occlusive disease involving the abdominal aorta and iliac arteries that is frequently associated with ischemic heart disease (IHD) in 40%–60% of patients, particularly those with diabetes mellitus [1, 2]. Several treatment strategies can be considered for patients with IHD complicated by Leriche syndrome. When coronary revascularization is the priority, particular caution is required during graft selection because the internal thoracic arteries (ITAs) may serve as collateral pathways to the lower limbs. We report the case of a patient with Leriche syndrome who presented with acute heart failure caused by IHD and was treated with on-pump total venous coronary artery bypass grafting (CABG).

## Case report

**Figure 1:**
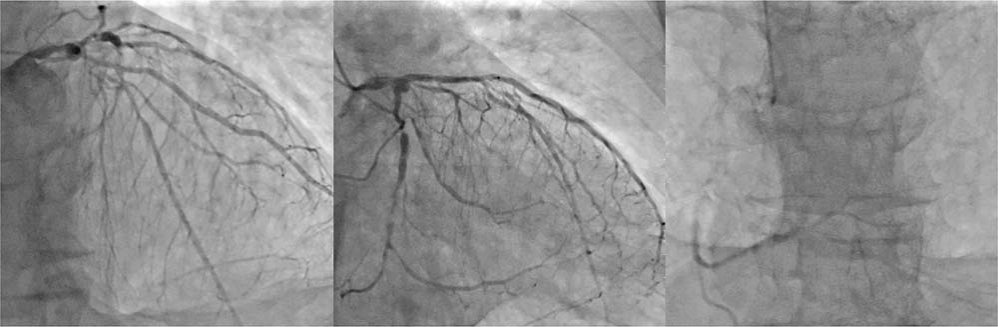
Preoperative coronary angiography. Preoperative coronary angiography image showing severe triple-vessel disease.

**Figure 2:**
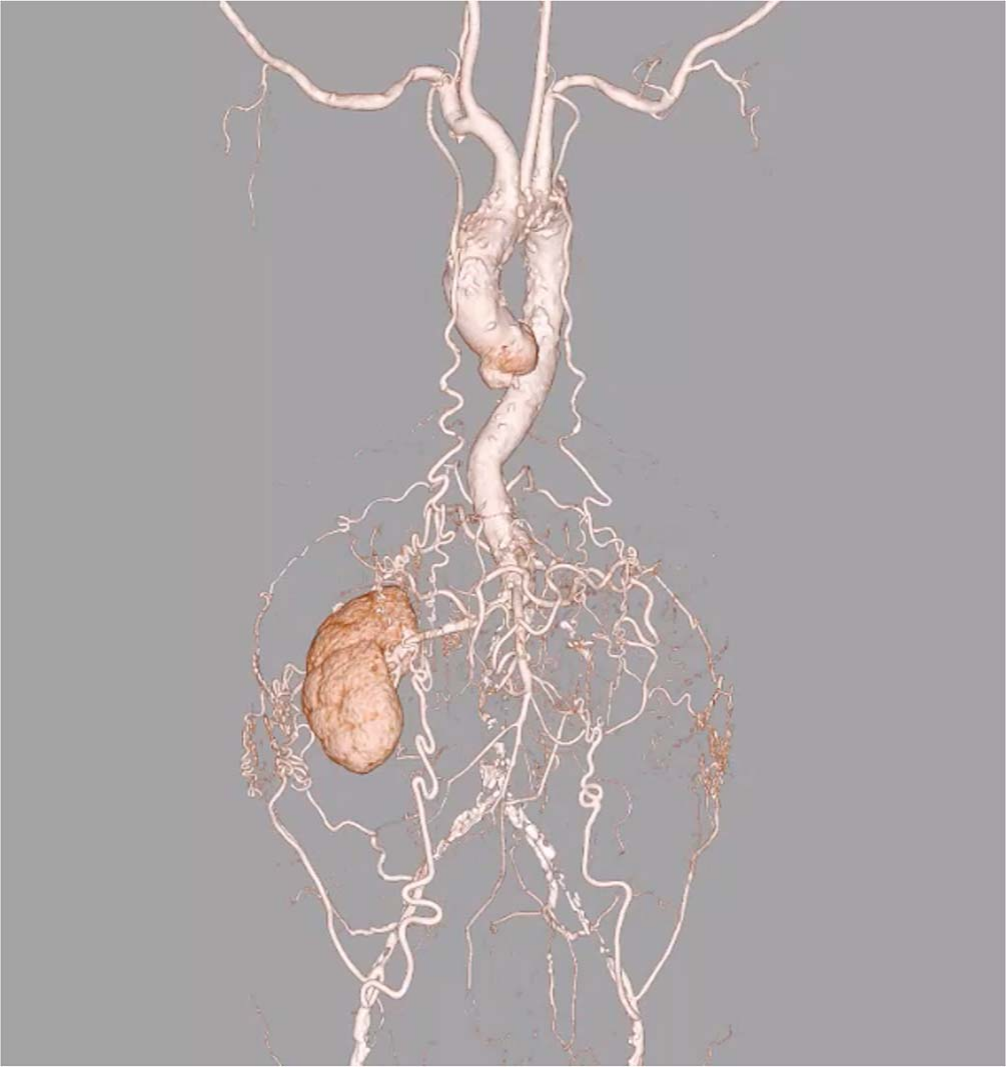
Preoperative 3D CT angiography of the entire vasculature. Preoperative 3D CT angiography image of the entire vasculature demonstrates that the ITAs are an important source of blood flow to the lower limbs.

**Figure 3:**
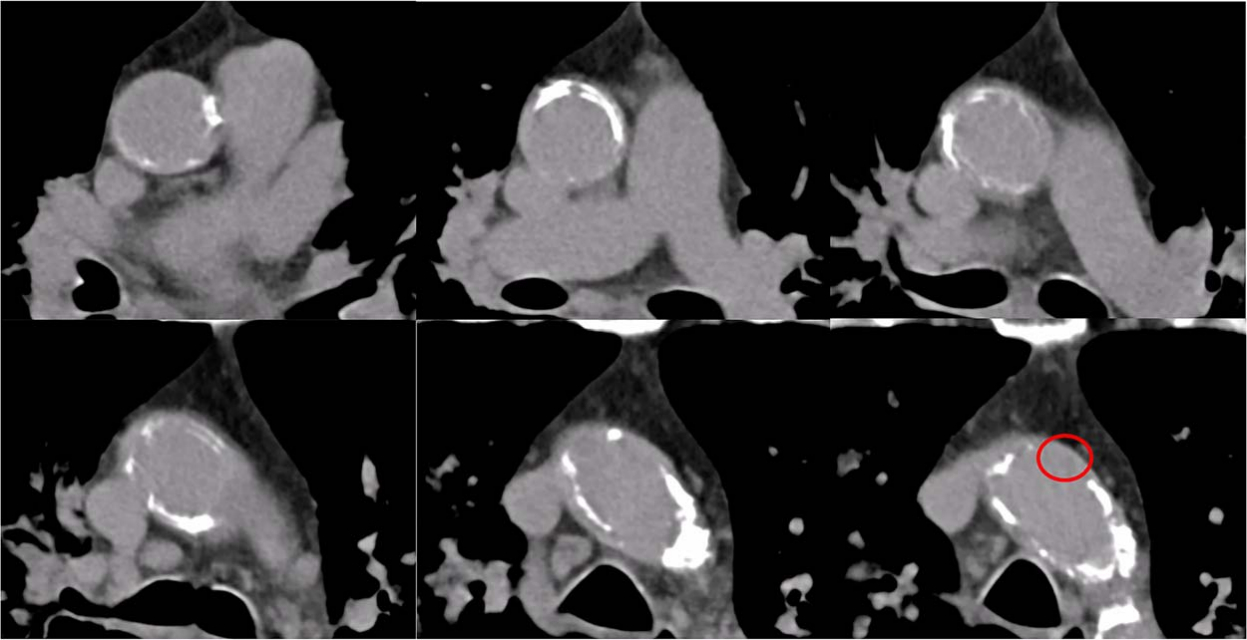
Preoperative contrast-enhanced CT of the ascending aorta. The ascending aorta is diffusely calcified. A region near the origin of the brachiocephalic artery is considered suitable for arterial cannulation. A proximal site above the fat band is also identified as appropriate for SVG anastomosis.

A 77-year-old man with chronic kidney disease was admitted to another hospital with heart failure and was diagnosed with IHD and triple vessel disease (Fig. 1). On examination, femoral pulses were absent, and angiography revealed occlusion of the abdominal aorta below the renal arteries. Therefore, he was transferred to our hospital for further management. Heart failure was stabilized with medical therapy, and semi-urgent surgery was planned. Because he had no symptoms in the lower extremities, elective performance of revascularization of the lower limbs was scheduled. The ITAs served as important collateral vessels to the lower limbs (Fig. 2); therefore, their use as grafts was considered a risk factor for lower limb ischemia. Transthoracic echocardiography revealed impaired systolic function with an ejection fraction of 42%, whereas contrast-enhanced computed tomography (CT) demonstrated diffuse calcification of the ascending aorta (porcelain aorta). However, an area near the origin of the brachiocephalic artery was considered suitable for arterial cannulation, and a proximal site above the fat band was identified as appropriate for saphenous vein graft (SVG) anastomosis (Fig. 3). Based on these findings, an on-pump total venous CABG strategy was selected, and surgery was performed on the second day after transfer.

**Figure 4:**
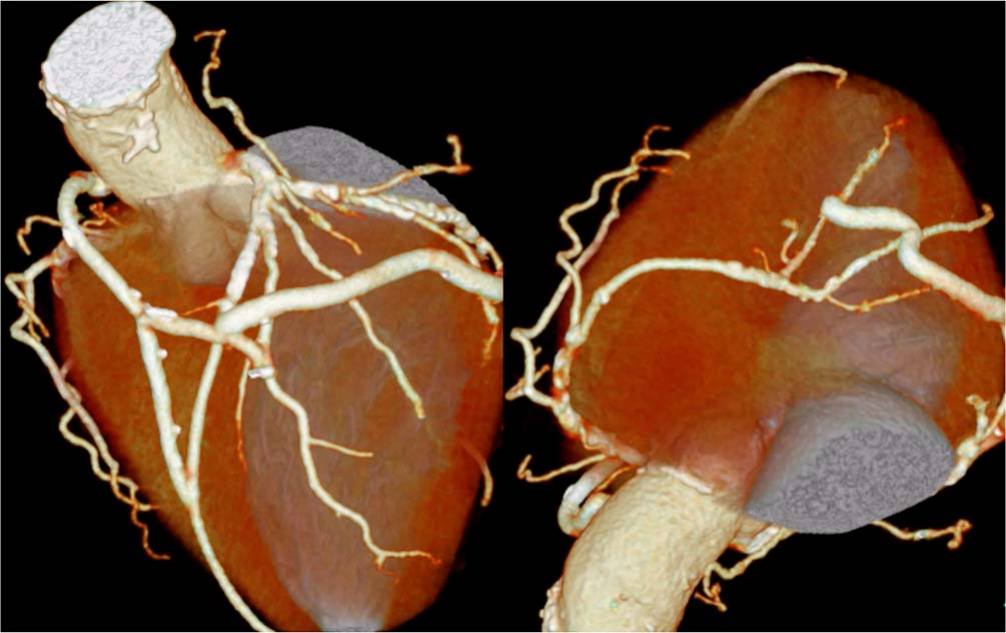
Postoperative coronary 3D CT angiography. Postoperative cardiac 3D CT image showing the final graft configuration. A SVG was anastomosed to the left anterior descending artery. Another SVG segment was joined end-to-side to construct a T-composite graft. This composite SVG was sequentially anastomosed to the diagonal branch, obtuse marginal branch, atrioventricular branch, and posterior descending branch. After revision because of torsion just distal to the diagonal anastomosis, the distal SVG segment was re-anastomosed end-to-side to the SVG connected to the diagonal branch, resulting in the final graft arrangement demonstrated in this image.

The patient underwent median sternotomy, and the non-touch SVG was harvested from the right leg. Cardiopulmonary bypass via the area around the origin of the brachiocephalic artery and the right atrium was established using the Seldinger technique. The proximal anastomosis of the SVG was performed on the right lateral wall of the ascending aorta using the Heartstring III Proximal Seal System (Maquet Cardiovascular LLC, Getinge AB, Rastatt, Germany), and exposure was facilitated by a stabilizer. The SVG was anastomosed to the left anterior descending artery, and another SVG segment was joined end-to-side to construct a T-composite graft. Then, the T-composite SVG was sequentially anastomosed to the diagonal branch, obtuse marginal branch, atrioventricular branch, and posterior descending branch. However, torsion caused narrowing of the SVG just distal to the diagonal anastomosis; therefore, a clip was applied, and the SVG was divided at that site. Then, the distal segment of the SVG was re-anastomosed end-to-side to the SVG that had been anastomosed to the diagonal branch (Fig. 4) (Video 1). After completion of the anastomoses, the patient was weaned from cardiopulmonary bypass, and surgery was completed. The postoperative course was uneventful, and the patient was transferred to another hospital for rehabilitation on postoperative day 28. At ~2 years after surgery, the patient was free from heart failure recurrence. Because symptoms of lower limb ischemia did not develop, revascularization of the lower limbs was not performed.

## Discussion

Leriche syndrome has been frequently associated with coronary artery disease [1, 2]. In the present case, diffuse calcification of the ascending aorta was observed. Therefore, we selected an on-pump total venous CABG strategy and achieved a favorable clinical course.

Management strategies for coronary and lower limb arterial diseases are broadly categorized as single-stage or staged revascularization. When a highly invasive single-stage approach, which consists of CABG combined with an aorto-femoral bypass, cannot be used, a staged revascularization strategy should be considered.

During staged revascularization, when coronary intervention is not urgently required, lower limb revascularization allows the use of the ITAs, which comprise the most valuable grafts for CABG, as reported by Ogoyama *et al.* [3]. This strategy could not be used for the present case because coronary revascularization was prioritized; however, it can be a reasonable option when clinically appropriate.

When coronary revascularization is the priority and restoration of lower limb inflow before revascularization is not possible, the use of ITAs should be avoided to preserve collateral perfusion to the lower limbs and, potentially, the spinal cord. Chouman et al. described postoperative paraplegia after CABG using ITAs in a patient with a subsequent diagnosis of Leriche syndrome, thus highlighting the importance of preoperative screening for aortoiliac occlusive disease [4]. In such situations, alternative conduits include the SVG and radial artery. Although the radial artery generally offers superior patency [5], factors such as age, renal dysfunction, and graft length should be considered when selecting the conduit. In this case, these factors led to the choice of a total venous CABG strategy.

Although the present case involved a porcelain aorta, a proximal anastomosis and arterial cannulation were feasible. When manipulation of the ascending aorta is contraindicated, the axillary artery is a potential option for proximal anastomosis, as reported by Ushioda *et al.*, who described the usefulness of the axillary artery as an inflow site for SVG [6]. In the present case, a limited area of the ascending aorta was suitable for proximal anastomosis. However, if such an area is not available, then the axillary artery may be a reasonable alternative.

## Supplementary Material

Operative_Video_rjaf1102

## Data Availability

All data supporting the findings of this case report are included within the article.
